# Impact of management of access to cardiac surgery in the Brazilian Unified Health System at a university hospital in Campinas: pre-post analysis, 2013-2019

**DOI:** 10.1590/S2237-96222025v34e20240222.en

**Published:** 2025-08-04

**Authors:** Silvia Thomas Antunes, Viviana Aparecida de Lima, Ivan Lira dos Santos, Rafaela Amadio Miguel, Gustavo Calado de Aguiar Ribeiro, Elisa Teixeira Mendes

**Affiliations:** 1 Pontifícia Universidade Católica de Campinas, Programa de Pós-Graduação Stricto Sensu em Ciências da Saúde, Campinas, SP, Brazil; 2 Pontifícia Universidade Católica de Campinas, Faculdade de Enfermagem, Campinas, SP, Brazil; 3 Hospital da Pontifícia Universidade Católica de Campinas, Faculdade de Ciências Médicas, Campinas, SP, Brazil

**Keywords:** Hospital Care, Cardiovascular Surgical Procedures, Unified Health System, Comprehensive Healthcare, Retrospective Studies, Atención Hospitalaria, Procedimientos Quirúrgicos Cardiovasculares, Sistema Único de Salud, Atención Integral de Salud, Estudios Retrospectivos

## Abstract

**Objective:**

To compare the pre- and post-implementation periods of a program to manage access to cardiac surgery for users of the SUS (Brazilian Unified Health System) in terms of surgical waiting time.

**Methods:**

This is an observational and retrospective study, which analyzed three years before and four years after the implementation of this program. Variables such as length of hospital stay, waiting time in the surgical queue and infection rate of users who underwent elective cardiovascular surgery in the SUS were evaluated.

**Results:**

There was a significant reduction in waiting times in the queue of 97.50 days in the pre-intervention period (95% confidence interval – 95%CI 79.00; 120.30) and 52.50 days (95%CI 44.40; 63.20) in the post-intervention period. There was a trend towards a reduction in the average hospital stay and an increase in the number of surgeries performed.

**Conclusion:**

The implementation of the management program reduced the waiting time for cardiac surgery in the SUS (Brazilian Unified Health System).

## Resumen

**Objetivo:**

Comparar los períodos pre y post implementación de un programa de gestión del acceso a cirugía cardíaca para usuarios del Sistema Único de Salud (SUS) en términos del tiempo de espera quirúrgica.

**Métodos:**

Se trata de un estudio observacional y retrospectivo, que analizó tres años antes y cuatro años después de la implementación de este programa. Se evaluaron variables como tiempo de internación hospitalaria, tiempo de espera en la fila quirúrgica y tasa de infección de usuarios sometidos a cirugía cardiovascular electiva en SUS.

**Resultados:**

Hubo una reducción significativa en los tiempos de espera en la cola de 97,50 días en el periodo preintervención (intervalo de confianza del 95% – IC95% 79,00; 120,30) y 52,50 días (IC95% 44,40; 63,20) en el periodo postintervención. Se observó una tendencia hacia una reducción de la estancia hospitalaria media y un aumento del número de cirugías realizadas.

**Conclusión:**

La implementación del programa de gestión redujo el tiempo de espera para cirugía cardíaca en SUS.

**Ethical aspects**

This research respected ethical principles, having obtained the following approval data::

Research Ethics Committee: Pontifícia Universidade Católica de Campinas

Opinion number: 3.663.083

Approval date: 30/11/2020

Certificate of Submission for Ethical Appraisal: 23253019.8.0000.5481

Informed Consent Form: Obtained from all participants prior to collection.

## Introduction

The aging of a large part of the world’s population has been noticeable in recent decades, and cardiovascular diseases are often present in this process. These diseases are responsible for almost a third of all deaths worldwide and for high social and economic costs (1). They affect the potentially active population (35-64 years old), causing death and sequelae and bringing great harm to the individual, family members and society (2). For people with cardiovascular diseases requiring surgical treatment, the time it takes to perform the surgery is crucial to the outcome of the cases (3).

The time between the surgical indication and its performance are indicators directly associated with the prognosis of cardiovascular diseases and the risks of surgical complications (3). Both influence the optimization of resources of the SUS (Brazilian Unified Health System) and the comprehensiveness of care. User access to more complex procedures presupposes the regulatory function of the SUS to optimize the population’s access to this resource and absorb such demand (4). In 2008, the Ministry of Health proposed the regulation of access and care flows within the SUS by institutional managers, through risk classification and access guarantee based on protocols (5).

Disparities were observed in access to cardiac surgeries in Brazil (6), although there is potential for improving care flows and reference centers (7). It was estimated that 3 million Brazilians were waiting to undergo some type of elective surgery in the SUS. In 2021, post-pandemic, this number reached 60 thousand in relation to cardiovascular procedures. In most cases, users remain in queues for years without any prospect of having the procedure performed. Most of the time, this scenario occurs due to a lack of good management of existing resources (8-10).

The management of hospital beds and of the surgical waiting list is strategic for optimizing resources within the hospital environment. The impact of access optimization is little evaluated in the literature, especially in surgical users. The objective of this study was to compare the pre- and post-implementation periods of a program to manage access to cardiac surgery for SUS users in terms of surgical waiting time.

## Methods

### Study design

This was a retrospective, longitudinal and observational study on waiting time in the healthcare queue after surgical indication, length of hospital stay and clinical outcomes of people with indication for elective cardiac surgery. The period “before and after” the implementation of the bed management system and user access management was compared.

### Context

The study took place at the Hospital of the Pontifical Catholic University of Campinas, whose service is philanthropic and university-based and has 339 beds. For SUS users, there are 204 beds (60.17%), 13 adult intensive care unit beds and four coronary intensive care unit beds. The latter is the only reference in cardiology, cardiovascular surgery and highly complex cardiac procedures in Campinas that is part of the Emergency and Urgency Network of the state of São Paulo.

The periods analyzed were pre-implementation of the bed management and access management system, from January 2013 to December 2015, and post-implementation, from February 2016 to December 2019. January was considered as the implementation period; therefore, there may be some inaccuracy when comparing 2016 with other years. 

### Participants

Users over 18 years of age who were referred for cardiovascular surgery and underwent the elective surgical procedure between 2013 and 2019 at the Hospital of the Pontifical Catholic University of Campinas were included.

Users who were admitted with an indication for emergency surgery and users of private health insurance treated at the service were excluded.

### Variables

During data collection, the medical records of all users who underwent elective cardiac surgery from 2013 to 2019 were evaluated, as well as information collected by the Hospital Infection Control Committee on surgical site infection of this hospital.

### Data sources and measurement

In addition to demographic variables (sex, age) and type of surgery performed, the indicators related to hospital admission investigated in the two study periods (pre- and post-intervention) were: time on the surgical waiting list in days, which described the time elapsed from the day of the surgical indication until its performance; average length of hospital stay in days; average time after admission to the coronary intensive care unit before discharge in days; fatality rate (%) in 90 days among those undergoing cardiac surgery and surgical site infection rate calculated by the ratio between the number of infections that met the criteria of the National Health Surveillance Agency; and the total number of cardiac surgeries performed in the period. This rate represented all cardiac surgeries performed by adult users of the hospital, including emergencies. 

Information was collected from outpatient and hospital medical and nursing records and during visits to hospitalized patients by the research team.

### Bias control

The analysis was based on secondary data. The severity of conditions and the impact on clinical outcome for the users could not be controlled.

### The intervention

**Figure 1:**
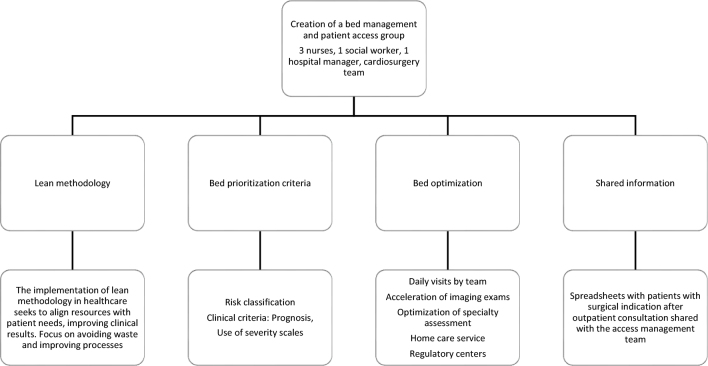
Intervention based on the creation of a bed and user access management group. Hospital of the Pontifical Catholic University of Campinas, 2013-2019

The intervention was related to create the “Bed Management and Patient Access Management” department, composed of 3 nurses, 1 social worker and hospital manager, in addition to cardiologists at times of reviewing/defining clinical criteria (Figure 1). 

The team relied on actions supported by the Lean methodology and logistical measures to optimize bed turnover indicators, reduce the average hospital stay and increase the institutional occupancy rate.

The Lean methodology was chosen for the intervention because, in the health area, this methodology has an approach focused on eliminating waste, that is, activities that do not add value to the user. Examples of these activities include long waiting times for care, exams, hospital stays, excessive travel by professionals or users due to poor organization and redundant processes such as filling out forms in different sectors.

This new team had total autonomy in managing hospitalizations, which previously was not centralized in this sector, often giving in to team demands and being performed in a disorganized manner. 

Criteria were established for: prioritization for bed occupancy, according to contingencies for emergency room overcrowding scenarios; and acceleration of pending cases to optimize bed turnover and have more vacancies for surgical calls. 

These actions were based on daily visits by a multidisciplinary team to the hospitalization units to resolve pending issues that would speed up discharge, such as accelerating imaging exams and optimizing specialty assessments (contact with the team’s medical coordinators).

### Other actions are detailed below.

- Activation of the home care service for cases that require discharge from hospital, interface with health service regulatory centers to transfer less complex cases and counter-referral of cases that have already been resolved.
- Receiving cases requiring highly complex resources from other institutions, regulated through the Health Service Offering Regulation Center of the state of São Paulo.
- Sharing, among the access management team, of spreadsheets with data on users with indications for cardiac surgery in outpatient consultations, and the subsequent summoning of these users for surgery and their classification according to clinical prioritization criteria, defined together with the cardiology specialty.

It should be noted that the intervention described is still in force today, as it was established with the objective of improving the process that is now consolidated in the health service, including expansion to other surgical indications.

### Study size

During 2013-2019, 865 elective cardiac surgeries performed by the SUS at the Hospital of the Pontifical Catholic University of Campinas were analyzed.

### Statistical methods

Sociodemographic and clinical variables were compared in the pre- and post-intervention periods. The categories were presented in proportions (%) and 95% confidence intervals and compared using Pearson’s chi-square test. Continuous variables (median and quartiles) were analyzed using the Mann-Whitney U test for two independent samples. The normality of continuous variables was analyzed using the Shapiro-Wilk test. The level of statistical significance considered was 5.0% (p-value<0.050).

The collected data were systematized using an Excel spreadsheet version 15.41. Statistical analyses were performed using Epi.Info software (Epidemiological Information software) version 7.2.2.6.

## Results

During the period of 2013-2019, 865 elective cardiac surgeries performed by the SUS at the Hospital of the Pontifical Catholic University of Campinas that met the inclusion criteria were analyzed. The main characteristics of this population were described in the two study periods (Table 1). The median age in the pre-implementation period was significantly higher than after the intervention (p-value<0.050). There was no considerable difference between surgical indications in the two periods. Myocardial revascularization with and without extracorporeal circulation, with and without grafts, was the most frequently performed surgery (71.09%), followed by valve replacements, cardiac plasty and prosthetics (17.91%) (Table 1).

**Table 1:** Comparison between users undergoing elective cardiac surgery in the pre- (2013-2015) and post-implementation (2016-2019) periods of the bed management and access management system. Hospital of the Pontifical Catholic University of Campinas, 2013-2019 (n=865)

Variables	Pre-implementation 2013-2015 (n=371)	Post-implementation 2016-2019 (n=494)	Total (n=865)	p-value^c^
**Age group** (years)				
18-39	15 (4,04)	26 (5.26)	41 (4.73)	0.002
40-59	58 (15.63)	93 (18.82)	151 (17.45)	
60-69	108 (29.11)	186 (37.65)	294 (33.98)	
≥70	190 (51.21)	189 (38.25)	379 (43.81)	
**Gender**				
Male	233 (62.80)	336 (68.01)	569 (65.78)	0.060
Female	138 (37.19)	158 (31.98)	296 (34.21)	
**Type of surgery**				
Myocardial revascularization^a^	254 (68.46)	361 (73.07)	615 (71.09)	0.110
Valve prostheses/plasties	74 (19.94)	81 (16.39)	155 (17.91)	
Aortic reconstruction/aneurysm	11 (2.96)	5 (1,01)	16 (1.84)	
Closure of atrial septal defect/ventricular septal defect	15 (4,04)	14 (2.83)	29 (3.35)	
Others^b^	17 (4.58)	33 (6.68)	50 (5.78)	

^a^Myocardial revascularization with and without extracorporeal circulation; ^b^Others: intracardiac tumor resection, multiple surgeries, ascending aorta surgery; ^c^Chi-square test on dichotomous variables and U Mann-Whitney test in continuous variables.

Differences in outcomes were found in the pre- and post-intervention periods (Table 2, Figure 1). A shorter time on the surgical waiting list was observed in the post-intervention period, of 52.50 days (median=18 days), compared to the previous period of 97.49 days (median=36 days) (p-value<0.001), reaching 1,923 days of waiting, a value corresponding to more than five years of waiting (1,923 divided by 365). The average length of hospital stay was shorter after the intervention; however, there was no statistical difference (Table 2).

**Table 2:** Comparison of waiting times, hospitalization and intensive care unit stays between the pre- (2013-2015) and post-intervention (2016-2019) periods using a 95% confidence interval (95%CI). Hospital of the Pontifical Catholic University of Campinas, 2013-2019 (n=865)

Indicators	Pre-intervention (2013-2015) (n=371)	Post-intervention (2016-2019) (n=494)	p-value^a^
Waiting time for surgery	97.50 (79.00; 120.30)	52.50 (44.40; 63.20)	<0.001
Average length of hospital stay (days)	12.60 (3.00; 52.00)	11.50 (3.0; 71.0)	0.100
Preoperative hospital stay (days)	3.0 (2.70; 3.40)	2.93 (2.60; 3.30)	0.410
Time between discharge from coronary intensive care unit and hospital discharge (days)	4.20 (3.70; 4.80)	3.98 (3.70; 4.40)	0.990

^a^Mann-Whitney U test for independent samples of continuous variables and Pearson’s chi-square test for proportions.

**Figure 2:**
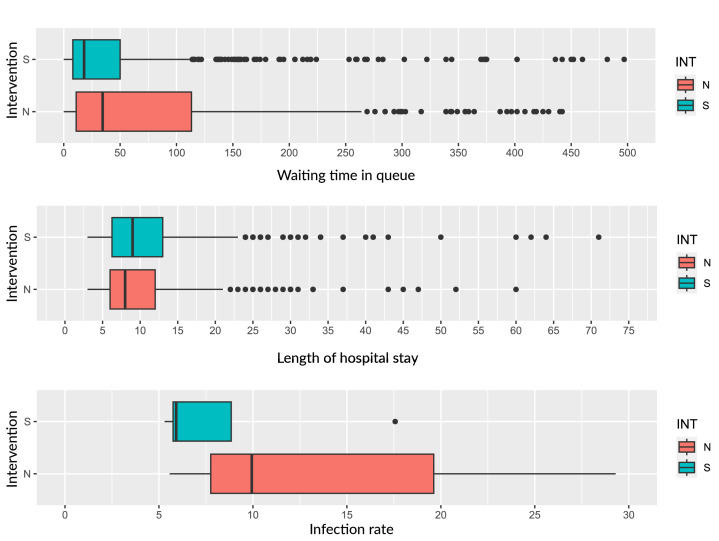
Comparison of clinical indicators and hospital flows of people with indication for elective cardiac surgery in the pre- and post-intervention period. Hospital of the Pontifical Catholic University of Campinas, 2013-2019

The surgical site infection rate throughout the period was 11.30%, with 14.90% in the pre-intervention period and 5.72% in the post-intervention period (p-value<0.001). In total, there were 33 deaths, with a 90-day fatality rate statistically similar in the pre- and post-intervention periods (p-value 0.440).

## Discussion

The substantial number of cardiac surgeries (865) performed in the SUS in seven years was analyzed. Myocardial revascularization was the most frequent type of elective surgery, like what happens in other public services (11-12).

After the intervention in the management of beds and user access, a considerable reduction in the waiting time between the indication and the performance of surgery was observed, with a median of 36 days in the pre-intervention period and 18 days in the post-intervention period. Interventions in bed management and user access were successful in reducing waiting times for procedures and significantly increasing surgical volume (13-17). The Lean methodology optimized resources in service management, from access to diagnosis and therapeutic procedures to healthcare flows and clinical outcomes (16).

Long waiting times for cardiac surgeries increase the risk of complications and unscheduled hospitalizations, especially for patients with a higher body mass index and heart failure (18,19).

This study evaluated the occurrence of deaths and the need for emergency surgery in people while waiting in queue for the proper intervention. Among 12,030 individuals, 500 (4.0%) experienced complications while waiting for planned surgery, including 104 deaths (21.0%) (20). The main consequences of prolonged waiting times were worse clinical outcomes, increased costs, and general dissatisfaction with the healthcare system (21).

There was a reduction in the length of hospital stay by more than one day, from 12.6 to 11.5 days (p-value 0.100), although without statistical significance. For 63,272 people who underwent coronary artery bypass grafting surgery in 175 hospitals served by the SUS, the average hospital stay was 12 days (22). The longest hospital stays were directly related to death and septic shock (23). For a day of hospitalization in a hospital with 300 beds, the equivalent was, on average, 49 new vacant beds (24).

There was a reduction in surgical site infection rates in this study, but it was observed that this data could not be directly related to the intervention. Reducing the average hospital stay and performing surgery at a more opportune time had the potential to reduce the risk of hospital infection (25). Predictors of surgical site infection in people undergoing cardiac surgery stood out, including comorbidities and surgical risk (26). Early surgical approach to users prevented clinical complications during the wait, which reduced the length of hospital stay and the risk of hospital infections (16,25-26).

Among the limitations of this study, the use of secondary data from medical records stood out. It was not possible to evaluate the severity score of users preoperatively to assess surgical risk. This limitation prevented an adequate assessment of the impact on user mortality. Recruiting people with long waiting times in the queue may have selected individuals in worse clinical conditions, which indicated a bias in the analysis of outcomes during hospitalization and limited the impact of the intervention.

This study demonstrated that simple adjustments to hospital resource management processes and access management can significantly reduce surgical waiting times for SUS users, with the potential to improve their prognosis. Prospective, multicenter studies are needed to more comprehensively assess the impact of this intervention on clinical outcomes.

## Data Availability

After publication, the data will be available on demand by contacting the authors.

## References

[1] Khushalani JS, Song S, Calhoun BH, Puddy RW, Kucik JE (2022). Preventing leading causes of death: systematic review of cost-utility literature. Am J Prev Med.

[2] Roth GA, Mensah GA, Johnson CO, Addolorato G, Ammirati E, Baddour LM (2019). Global burden of cardiovascular diseases and risk factors, 1990-2019: update from the GBD 2019 Study. J Am Coll Cardiol.

[3] Neves J, Pereira H, Uva MS, Gavina C, Moreira AL, Loureiro MJ (2015). Portuguese Society of Cardiothoracic and Vascular Surgery/Portuguese Society of Cardiology recommendations for waiting times for cardiac surgery. Rev Port Cardiol.

[4] Oliveira CCRB, Silva EAL, Souza MKB (2021). Referral and counter-referral for the integrality of care in the Health Care Network. Physis.

[5] Brasil (2008). Portaria MS/GM nº 1.559/2008. Institui a Política Nacional de Regulação do Sistema Único de Saúde (SUS).

[6] Viacava F, Porto S, Laguardia J, Moreira RS, Ugá MAD (2012). Diferenças regionais no acesso a cirurgia cardiovascular no Brasil, 2002-2010. Cien Saude Colet.

[7] Tiwari KK, Grapsa J, Laudari S, Pazdernik M, Vervoort D (2021). Challenges and possibilities of developing cardiac surgery in a peripheral hospital of low- and middle-income countries. Perfusion.

[8] Cavalcanti RP, Cruz DF, Padilha WWN (2018). Desafios da regulação assistencial na organização do Sistema Único de Saúde. R Bras Cien Saúde.

[9] Freire MP, Louvison M, Feuerwerker LCM, Chioro A, Bertussi D (2020). Regulação do cuidado em redes de atenção: importância de novos arranjos tecnológicos. Saúde Soc.

[10] Félix P (2021). Brasil tem fila de 60 mil à espera de cirurgias cardiovasculares [Internet].

[11] Raffa C, Malik AM, Pinochet LHC (2017). Análise das variáveis do ambiente interno no gerenciamento de leitos em organizações hospitalares privadas: aplicação do software NVIVO. RAHIS.

[12] Piegas LS, Bittar OJNV, Haddad N (2009). Cirurgia de revascularização miocárdica: resultados do Sistema Único de Saúde. Arq Bras Cardiol.

[13] Rocha ASC, Pittella FJM, Lorenzo AR, Barzan V, Colafranceschi AS, Brito JOR (2012). A idade influencia os desfechos em pacientes com idade igual ou superior a 70 anos submetidos à cirurgia de revascularização miocárdica isolada. Rev Bras Cir Cardiovasc.

[14] Hoefsmit PC, Schretlen S, Burchell G, van den Heuvel J, Bonjer J, Dahele M (2022). Can quality improvement methodologies derived from manufacturing industry improve care in cardiac surgery?. A systematic review.

[15] Watling A, Doucet J, Zohrabi M, Fedirko J, Hassan A, Lutchmedial S (2020). Impact on cardiac surgery volume of a comprehensive partnership with Integrated Health Solutions. Can J Surg.

[16] Mason SE, Nicolay CR, Darzi A (2015). The use of Lean and Six Sigma methodologies in surgery: a systematic review. Surgeon.

[17] Hallam CRA, Contreras C (2018). Lean healthcare: scale, scope and sustainability. Int J Health Care Qual Assur.

[18] Sun LY, Eddeen AB, Wijeysundera HC, Mamas MA, Tam DY, Mesana TG (2021). Derivation and validation of a clinical model to predict death or cardiac hospitalizations while on the cardiac surgery waitlist. CMAJ.

[19] Head SJ, Costa BR, Beumer B, Stefanini GG, Alfonso F, Clemmensen PM (2017). Adverse events while awaiting myocardial revascularization: a systematic review and meta-analysis. Eur J Cardiothorac Surg.

[20] Sobolev BG, Fradet G, Kuramoto L, Rogula B (2013). The occurrence of adverse events in relation to time after registration for coronary artery bypass surgery: a population-based observational study. J Cardiothorac Surg.

[21] McIntyre D, Chow CK (2020). Waiting time as an indicator for health services under strain: a narrative review. Inquiry.

[22] Moraes CMT, Corrêa LM, Procópio RJ, Carmo GALD, Navarro TP (2022). Tools and scores for general and cardiovascular perioperative risk assessment: a narrative review. Rev Col Bras Cir.

[23] Koerich C, Lanzoni GMM, Erdmann AL (2016). Factors associated with mortality in patients undergoing coronary artery bypass grafting. Rev Lat Am Enfermagem.

[24] Ceballos-Acevedo TM, Velásquez-Restrepo PA, Jaén-Posada JS (2014). Duración de la estancia hospitalaria. Metodologías para su intervención. Rev Gerenc Polit Salud.

[25] Ferreira GB, Donadello JCS, Mulinari LA (2020). Healthcare-associated infections in a cardiac surgery service in Brazil. Braz J Cardiovasc Surg.

[26] Andrade LS, Siliprandi EMO, Karsburg LL, Berlesi FP, Carvalho OLF, Rosa DS (2019). “Bundle” de prevenção de sítio cirúrgico em cirurgia cardíaca. Arq Bras Cardiol.

