# Supplementary material for: Impact of management of access to cardiac surgery in the Brazilian Unified Health System at a university hospital in Campinas: pre-post analysis, 2013-2019
Source: Epidemiol Serv Saude. 2025 Aug 4;34:e20240222. doi: 10.1590/S2237-96222025v34e20240222.en (PMC12338985; doi:10.1590/S2237-96222025v34e20240222.en)
Supplement: Translation

# Translation: Impacto da gestão do acesso à cirurgia cardíaca no Sistema Único de Saúde em hospital universitário de Campinas: análise do tipo antes e depois, 2013-2019

**DOI:** 10.1590/S2237-96222025v34e20240222.pt

**Authors:** Silvia Thomas Antunes, Viviana Aparecida de Lima, Ivan Lira dos Santos, Rafaela Amadio Miguel, Gustavo Calado de Aguiar Ribeiro, Elisa Teixeira Mendes

**Affiliations:** 1 Pontifícia Universidade Católica de Campinas, Programa de Pós-Graduação Stricto Sensu em Ciências da Saúde, Campinas, SP, Brasil; 2 Pontifícia Universidade Católica de Campinas, Faculdade de Enfermagem, Campinas, SP, Brasil; 3 Hospital da Pontifícia Universidade Católica de Campinas, Faculdade de Ciências Médicas, Campinas, SP, Brasil

**Keywords:** Assistência Hospitalar, Procedimentos Cirúrgicos Cardiovasculares, Sistema Único de Saúde, Assistência Integral à Saúde, Estudos Retrospectivos

## Abstract

**Objetivo:**

Comparar os períodos pré e pós-implantação de um programa de gestão do acesso à cirurgia cardíaca dos usuários do Sistema Único de Saúde (SUS) no tempo de fila cirúrgica.

**Métodos:**

Trata-se de estudo observacional e retrospectivo, que analisou três anos antes e quatro anos após a implantação desse programa. Foram avaliadas variáveis de tempo de internação, tempo de espera na fila cirúrgica e taxa de infecção dos usuários que realizaram cirurgia cardiovascular eletivamente no SUS.

**Resultados:**

Houve redução significativa nos tempos de espera na fila de 97,50 dias no período pré (intervalo de confiança de 95% – IC95% 79,00; 120,30) e de 52,50 dias (IC95% 44,40; 63,20) no período pós-intervenção. Observou-se tendência de redução da média de permanência hospitalar e de aumento do número de cirurgias realizadas.

**Conclusão:**

A implantação do programa da gestão diminuiu o tempo de fila de espera à cirurgia cardíaca no SUS.

**Aspectos éticos**

A presente pesquisa respeitou os princípios éticos, obtendo os seguintes dados de aprovação: :

Comitê de ética em pesquisa: Pontifícia Universidade Católica de Campinas

Número do parecer: 3.663.083

Data de aprovação: 30/11/2020

Certificado de apresentação de apreciação ética: 23253019.8.0000.5481

Registro do consentimento livre e esclarecido: Obtido de todos os participantes antes da coleta.

## Introdução

O envelhecimento de boa parte da população mundial é perceptível nas últimas décadas, e as doenças cardiovasculares, muitas vezes, se fazem presentes nesse processo. Estas são responsáveis por quase um terço de todas as mortes no mundo e por elevados custos sociais e econômicos (1). Tais doenças acometem a população potencialmente ativa (35-64 anos), causando morte e sequelas e desencadeando grandes danos ao indivíduo, aos familiares e à sociedade (2). Para as pessoas com doenças cardiovasculares com indicação de tratamento cirúrgico, o tempo para realização da cirurgia é crucial para definição do desfecho dos casos (3).

O tempo entre a indicação cirúrgica e a sua realização são indicadores diretamente associados ao prognóstico das doenças cardiovasculares e dos riscos de complicações cirúrgicas (3). Ambos influenciam a otimização de recursos do Sistema Único de Saúde (SUS) e a integralidade do cuidado. O acesso do usuário a procedimentos de maior complexidade pressupõe a função regulatória do SUS para otimizar o acesso da população a esse recurso e absorver tal demanda (4). Em 2008, o Ministério da Saúde propôs a regulamentação do acesso e dos fluxos assistenciais no âmbito do SUS pelos gestores institucionais, por meio de classificação de risco e garantia de acesso com base em protocolos (5).

Observaram-se disparidades no acesso a cirurgias cardíacas no Brasil (6), embora com a possibilidade de aperfeiçoamento dos fluxos assistenciais e dos centros de referência (7). Estimou-se que 3 milhões de brasileiros aguardavam em filas para realização de algum tipo de cirurgia eletiva no SUS. Em 2021, no pós-pandemia, esse número chegou a 60 mil em relação aos procedimentos cardiovasculares. Na maior parte dos casos, os usuários permanecem por anos nas filas sem nenhuma previsão de realização do procedimento. Na maioria das vezes, tal cenário ocorre por falta da boa gestão dos recursos existentes (8-10).

A gestão dos leitos nas organizações hospitalares e da fila de espera cirúrgica é estratégica para otimização de recursos no âmbito hospitalar. O impacto da otimização do acesso é pouco avaliado na literatura, especialmente nos usuários cirúrgicos. O objetivo deste estudo foi comparar os períodos pré e pós-implantação de um programa de gestão do acesso à cirurgia cardíaca dos usuários do SUS no tempo de fila cirúrgica.

## Métodos

### Delineamento do estudo

Tratou-se de estudo retrospectivo, longitudinal e observacional sobre tempo de espera na fila após a indicação cirúrgica, tempo de internação e desfechos clínicos de pessoas com indicação de cirurgia cardíaca eletiva. Comparou-se o período “antes e depois” da implantação do sistema de gerenciamento de leitos e gestão do acesso do usuário.

### Contexto

O estudo ocorreu no Hospital da Pontifícia Universidade Católica de Campinas, cujo serviço é filantrópico e universitário e conta com 339 leitos. Para os usuários do SUS, são 204 leitos (60,17%), 13 leitos de unidade de terapia intensiva adulto e quatro de unidade de terapia intensiva coronariana. Esta última é a única referência em cardiologia, cirurgia cardiovascular e procedimentos cardíacos de alta complexidade em Campinas que integra a Rede de Urgência e Emergência do estado de São Paulo.

Os períodos analisados foram a pré-implantação do sistema de gerenciamento de leitos e gestão do acesso, de janeiro de 2013 a dezembro de 2015, e a pós-implantação, de fevereiro de 2016 a dezembro de 2019. Considerou-se janeiro como o período de implantação; por conta disso, pode ocorrer alguma imprecisão em relação à comparação de 2016 com os outros anos. 

### Participantes

Foram incluídos os usuários maiores de 18 anos que tiveram indicação de cirurgia cardiovascular e realizaram o procedimento cirúrgico eletivo no período 2013-2019 no Hospital da Pontifícia Universidade Católica de Campinas.

Excluíram-se os usuários que foram internados com indicação de cirurgia de urgência e os usuários de convênios particulares atendidos no serviço.

### Variáveis

Na coleta de dados, foram avaliados os prontuários de todos os usuários que realizaram cirurgia cardíaca eletiva de 2013 a 2019, bem como as informações coletadas pela Comissão de Controle de Infecção Hospitalar do hospital sobre infecção no sítio cirúrgico.

### Fontes de dados e mensuração

Além das variáveis demográficas (sexo, idade) e do tipo de cirurgia realizada, os indicadores referentes à internação hospitalar investigados nos dois períodos de estudo (pré e pós-intervenção) foram: tempo na fila de espera cirúrgica em dias, que descrevia o tempo percorrido a partir do dia da indicação cirúrgica até a sua realização; tempo médio de permanência hospitalar em dias; tempo médio após internação na unidade de terapia intensiva coronariana antes da alta em dias; taxa de letalidade (%) em 90 dias entre os submetidos à cirurgia cardíaca e taxa de infecção do sítio cirúrgico calculada pela razão entre o número de infecções que preenchiam os critérios da Agência Nacional de Vigilância Sanitária; e o total de cirurgias cardíacas realizadas no período. Essa taxa representava todas as cirurgias cardíacas dos usuários adultos do hospital, sendo também incluídas as urgências. 

Informações foram coletadas em prontuários médicos e de enfermagem ambulatoriais, hospitalares e durante as visitas às pessoas internadas por parte da equipe de pesquisa.

### Controle de viés

A análise baseou-se em dados secundários. Não foi possível controlar a gravidade dos usuários e o impacto no desfecho clínico.

### A intervenção

A intervenção foi principalmente criar o departamento de “Gerenciamento de Leitos e Gestão do Acesso do Paciente” composto por 3 enfermeiros, 1 assistente social e gestor do hospital, além de médicos cardiologistas em momentos de revisão/definição de critérios clínicos (Figura 1). 

**Figura 1:** Intervenção com base na criação de grupo de gestão de leitos e acesso ao usuário. Hospital da Pontifícia Universidade Católica de Campinas, 2013-2019

A equipe se baseou em ações com suporte na metodologia Lean e medidas logísticas para otimizar os indicadores de giro de leito, reduzir a média de permanência hospitalar e aumentar a taxa de ocupação institucional.

A metodologia Lean foi escolhida para a intervenção, pois, na área da saúde, tal metodologia tem abordagem focada na eliminação de desperdícios, ou seja, atividades que não agregam valor ao usuário. São exemplos dessas atividades: longos tempos de espera para atendimento, exames, tempo de internação, deslocamentos excessivos de profissionais ou usuários devido à má organização e processos redundantes como o preenchimento de formulários em diferentes setores.

Essa nova equipe contou com autonomia total na gestão das internações, que anteriormente não se encontrava centralizada nesse setor, muitas vezes cedendo a demandas de equipes e de forma desorganizada. 

Foram estabelecidos critérios de: priorização para a ocupação dos leitos, conforme contingências para os cenários de superlotação dos prontos-socorros; e aceleração de pendências para otimizar o giro de leitos e contar com mais vagas para a convocação cirúrgica. 

Tais ações basearam-se em visitas diárias por equipe multiprofissional nas unidades de internação para resolver pendências que agilizassem a desospitalização, a exemplo da aceleração de exames de imagem e da otimização da avaliação de especialidades (contato com os coordenadores médicos das equipes).

Outras ações são detalhadas a seguir.

- Acionamento do serviço de atendimento domiciliar para os casos passíveis de desospitalização, interface com as centrais reguladoras de serviços de saúde para transferência dos casos de menor complexidade e contrarreferência dos casos já resolvidos.
- Recebimento de casos necessitados dos recursos da alta complexidade de outras instituições, regulados por meio da Central de Regulação de Ofertas de Serviços de Saúde do estado de São Paulo.
- Compartilhamento, entre a equipe da gestão de acesso, de planilhas com dados dos usuários com indicação de cirurgia cardíaca em consulta ambulatorial, e a consequente convocação desses usuários para cirurgia e a classificação destes conforme os critérios clínicos de priorização, definidos em conjunto com a especialidade de cardiologia.

Salienta-se que a intervenção descrita está em vigência até o presente momento, pois foi estabelecida com o objetivo assistencial de melhorar o processo que hoje é consolidado no serviço de saúde, inclusive com ampliação para as demais indicações cirúrgicas.

### Tamanho do estudo

No período 2013-2019, foram analisadas 865 cirurgias cardíacas eletivas realizadas pelo SUS no Hospital da Pontifícia Universidade Católica de Campinas.

### Métodos estatísticos

As variáveis sociodemográficas e clínicas foram comparadas nos períodos pré e pós-intervenção. As categorias foram apresentadas em proporções (%) e intervalos de confiança de 95% e comparadas pelo teste qui-quadrado de Pearson. As variáveis contínuas (mediana e quartis) foram analisadas por meio do teste U de Mann-Whitney para duas amostras independentes. A normalidade das variáveis contínuas foi analisada com o teste Shapiro-Wilk. O nível de significância estatística considerado foi 5,0% (p-valor<0,050).

Os dados coletados foram sistematizados utilizando-se planilha Excel versão 15.41. As análises estatísticas foram realizadas por meio do programa Epi.Info (Epidemiological Information software) versão 7.2.2.6.

## Resultados

No período 2013-2019, foram analisadas 865 cirurgias cardíacas eletivas realizadas pelo SUS no Hospital da Pontifícia Universidade Católica de Campinas que se enquadram nos critérios de inclusão. Foram descritas as principais características dessa população nos dois períodos de estudo (Tabela 1). A idade mediana no período pré-implantação foi significativamente maior do que após a intervenção (p-valor<0,050). Não houve diferença considerável entre as indicações cirúrgicas nos dois períodos. A revascularização miocárdica com e sem circulação extracorpórea, com e sem enxertos, foi a cirurgia realizada com maior frequência (71,09%), seguida por trocas, plastia e próteses valvares (17,91%) (Tabela 1).

**Tabela 1:** Comparação entre os usuários submetidos à cirurgia cardíaca eletiva nos momentos pré (2013-2015) e pós-implantação (2016-2019) do sistema de gerenciamento de leitos e gestão do acesso. Hospital da Pontifícia Universidade Católica de Campinas, 2013-2019 (n=865)

Variáveis	Pré-implantação 2013-2015 (n=371)	Pós-implantação 2016-2019 (n=494)	Total (n=865)	p-valor^c^
**Faixa etária** (anos)				
18-39	15 (4,04)	26 (5,26)	41 (4,73)	0,002
40-59	58 (15,63)	93 (18,82)	151 (17,45)	
60-69	108 (29,11)	186 (37,65)	294 (33,98)	
≥70	190 (51,21)	189 (38,25)	379 (43,81)	
**Sexo**				
Masculino	233 (62,80)	336 (68,01)	569 (65,78)	0,060
Feminino	138 (37,19)	158 (31,98)	296 (34,21)	
**Tipo de cirurgia**				
Revascularização miocárdica^a^	254 (68,46)	361 (73,07)	615 (71,09)	0,110
Próteses/plastias valvares	74 (19,94)	81 (16,39)	155 (17,91)	
Reconstrução/aneurisma aorta	11 (2,96)	5 (1,01)	16 (1,84)	
Fechamento comunicação interatrial/comunicação interventricular	15 (4,04)	14 (2,83)	29 (3,35)	
Outras^b^	17 (4,58)	33 (6,68)	50 (5,78)	

^a^Revascularização miocárdica com e sem circulação extracorpórea; ^b^Outras: ressecção tumor intracardíaco, cirurgias múltiplas, cirurgia aorta ascendente; ^c^Teste qui-quadrado em variáveis dicotômicas e teste U de Mann-Whitney em contínuas.

Foram encontradas diferenças de desfechos nos períodos pré e pós-intervenção (Tabela 2, Figura 1). Observou-se menor tempo na fila de espera cirúrgica no período pós-intervenção, de 52,50 dias (mediana=18 dias), comparado ao período prévio de 97,49 dias (mediana=36 dias) (p-valor<0,001), chegando a 1.923 dias de espera, valor correspondente a mais de cinco anos de fila (1.923 dividido por 365). O tempo médio de internação foi menor após a intervenção, entretanto não houve diferença estatística (Tabela 2).

**Tabela 2:** Comparação dos tempos de espera, internação e unidade de terapia intensiva entre os períodos pré (2013-2015) e pós-intervenção (2016-2019) por meio de intervalo de confiança de 95% (IC95%). Hospital da Pontifícia Universidade Católica de Campinas, 2013-2019 (n=865)

Indicadores	Pré-intervenção (2013-2015) (n=371)	Pós-intervenção (2016-2019) (n=494)	p-valor^a^
Tempo na fila de espera para cirurgia	97,50 (79,00; 120,30)	52,50 (44,40; 63,20)	<0,001
Tempo médio de internação (dias)	12,60 (3,00; 52,00)	11,50 (3,0; 71,0)	0,100
Tempo hospitalar pré-operatório (dias)	3,0 (2,70; 3,40)	2,93 (2,60; 3,30)	0,410
Tempo entre alta unidade de terapia intensiva coronariana e alta hospitalar (dias)	4,20 (3,70; 4,80)	3,98 (3,70; 4,40)	0,990

^a^Teste U de Mann-Whitney para amostras independentes de variáveis contínuas e teste qui-quadrado de Pearson para proporções.

**Figura 2:** Comparação de indicadores clínicos e fluxos hospitalares de pessoas com indicação de cirurgia cardíaca eletiva no período pré e pós-intervenção. Hospital da Pontifícia Universidade Católica de Campinas, 2013-2019

A taxa de infecção do sítio cirúrgico em todo período foi 11,30%, sendo 14,90% no período pré-intervenção e 5,72% no pós (p-valor<0,001). No total, foram 33 óbitos, com taxa de letalidade em 90 dias estatisticamente semelhante nos períodos pré e pós-intervenção (p-valor 0,440).

## Discussão

Analisou-se o número significativo de cirurgias cardíacas (865) realizadas no SUS em sete anos. A revascularização do miocárdio foi o tipo de cirurgia eletiva mais frequente, como encontrado em outros serviços públicos (11-12).

Após a intervenção na gestão dos leitos e do acesso dos usuários, observou-se redução considerável do tempo de espera entre a indicação e a realização da cirurgia, com mediana que foi de 36 dias, no período pré-intervenção, para 18 dias no período pós. As intervenções na gestão de leitos e do acesso dos usuários apresentaram sucesso na redução do tempo de espera por procedimentos e no aumento significativo no volume cirúrgico (13-17). A metodologia Lean otimizou recursos na gestão de serviços, desde o acesso ao diagnóstico e aos procedimentos terapêuticos até os fluxos assistenciais e desfechos clínicos (16).

Os longos períodos de espera por cirurgias cardíacas aumentam risco de intercorrências, internações não programadas, especialmente para os usuários com maior índice de massa corpórea e insuficiência cardíaca (18,19).

Este estudo avaliou a ocorrência de óbitos e a necessidade de cirurgia de emergência nas pessoas durante a espera em filas. Entre 12.030 indivíduos, 500 (4,0%) apresentaram intercorrência enquanto esperavam por cirurgia planejada, entre eles 104 óbitos (21,0%) (20). As principais consequências dos tempos de espera prolongados foram desfechos clínicos piores, aumento dos custos e insatisfação geral com o sistema de saúde (21).

Houve redução no tempo de internação hospitalar em mais de um dia, de 12,6 para 11,5 dias (p-valor 0,100), porém sem significância estatística. Para 63.272 pessoas submetidas à cirurgia de revascularização do miocárdio em 175 hospitais, atendidos pelo SUS, a média de permanência hospitalar foi 12 dias (22). Os maiores tempos de internação hospitalar tiveram relação direta com óbito e choque séptico (23). Para um dia de internação em hospital com 300 leitos, a equivalência era, em média, a 49 novos leitos vagos (24).

Ocorreu a redução nas taxas de infecção do sítio cirúrgico neste estudo, porém observou-se que esse dado não pode ser diretamente relacionado à intervenção. A redução da média de permanência hospitalar e a cirurgia em momento mais oportuno tiveram potencial de reduzir o risco de infecção hospitalar (25). Destacaram-se os preditores de infecção do sítio cirúrgico em pessoas submetidas à cirurgia cardíaca, entre eles as comorbidades e o risco cirúrgico (26). A abordagem cirúrgica precoce de usuários pôde evitar intercorrências clínicas durante a espera, o que diminuiu o tempo de internação e o risco de infecções hospitalares (16,25-26).

Entre as limitações deste estudo, destacou-se a utilização de dados secundários de prontuários médicos. Não foi possível avaliar o escore de gravidade de usuários no pré-operatório para avaliação de risco cirúrgico. Essa limitação impediu a adequada avaliação do impacto na mortalidade dos usuários. A captação de pessoas com longo tempo de espera na fila pode ter selecionado indivíduos em piores condições clínicas, o que indicou um viés na análise dos desfechos durante a internação e limitou o impacto da intervenção.

Este estudo demonstrou que ajustes simples nos processos de gerenciamento de recursos hospitalares e na gestão do acesso podem reduzir significativamente o tempo de espera cirúrgica para usuários do SUS, com potencial para melhorar o prognóstico destes. Estudos prospectivos e multicêntricos são necessários para avaliar, de forma mais abrangente, o impacto dessa intervenção nos desfechos clínicos.

## Data Availability

Após a publicação, os dados estarão disponíveis sob demanda aos autores.
